# Mucinous Adenocarcinoma of Gallbladder: An Incidental Finding of Acute Cholecystitis With Cholelithiasis

**DOI:** 10.7759/cureus.41089

**Published:** 2023-06-28

**Authors:** Fahim Soukhak, Tanapong J Shugan, Olivier Urayeneza

**Affiliations:** 1 Surgery, Ross University School of Medicine, Los Angeles, USA; 2 Family Medicine, Doctors Medical Center, Modesto, USA; 3 General Surgery, California Hospital Medical Center, Los Angeles, USA

**Keywords:** incidental gall bladder carcinoma, mucinous adenocarcinomas, laporoscopic cholecystectomy, gall bladder malignancy, acute calculus cholecystitis

## Abstract

The biliary tract's most frequent malignancy is gallbladder cancer (GBC). Due to the growing number of laparoscopic cholecystectomy procedures, the majority of GBC cases in the United States are diagnosed as incidental findings. Roughly 5% of the reported cases of gallbladder adenocarcinomas are of the mucinous type. Mucinous adenocarcinoma tumors contain more than 50% of mucin materials and are clinically characterized by poor prognosis. Initial presentation can mimic acute cholecystitis or cholelithiasis; meanwhile, histological findings post-cholecystectomy are the main diagnostic criteria of mucinous adenocarcinoma of the gallbladder. This case presents a patient who has been diagnosed with cholelithiasis and cholecystitis and subsequently underwent laparoscopic cholecystectomy. Histopathological examination of the gallbladder specimen was consistent with incidental gallbladder adenocarcinoma, the mucinous subtype to be specific.

## Introduction

Gallbladder cancer (GBC) is the most common malignancy of the biliary tract and adenocarcinomas are the most common type of GBC [1]. With the increase in laparoscopic cholecystectomy procedures, most GBCs are diagnosed incidentally in the United States and reported in around 0.25%-0.7% of specimens [1]. Roughly 5% of the reported cases of gallbladder adenocarcinomas are of the mucinous type. Mucinous adenocarcinoma tumors contain more than 50% of mucin components and are clinically characterized by poor prognosis. Initial presentation can mimic acute cholecystitis or cholelithiasis and histological findings post-cholecystectomy are the main diagnostic criteria of mucinous adenocarcinoma of the gallbladder [2]. Incidental gallbladder cancer (IGBC) is typically early-stage cancer as opposed to non-incidental; therefore, early surgical management confers increased survival benefits [3]. The surgical technique of choice is an Immediate Re-Resection (IRR) with wedge resection of the gallbladder bed and locoregional lymphadenectomy of the hepatoduodenal ligament. However, this is the treatment of choice if the T stage is above T1a. Otherwise, if it is T1a, cholecystectomy is sufficient and there is no need for IRR. This case report describes a patient with an incidental pathology finding of gallbladder cancer after laparoscopic cholecystectomy for acute calculous cholecystitis and cholelithiasis.

## Case presentation

The patient was an 84-year-old Hispanic male, with a history of hypertension and prostate surgery, who presented with epigastric pain for two days. The pain worsened after meals and was accompanied by nausea. The patient denied vomiting, fever, and weight loss. On physical examination, the patient had epigastric tenderness on palpation, negative Murphy’s sign, and no scleral icterus. Notable labs included total Bilirubin of 1.6 mg/dL and WBC 11.9 x10^9^/L but normal liver function tests.

**Figure 1:**
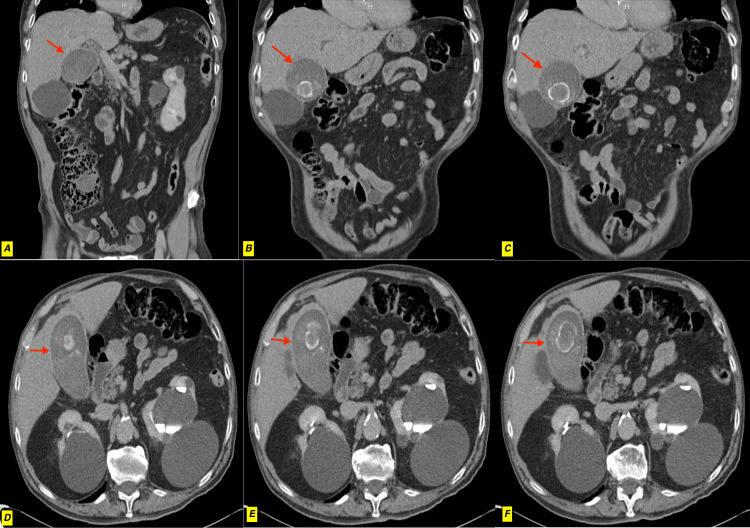
CT abdomen images CT scan of the abdomen showed a distended gallbladder (A) containing gallstones (B, C, D, E, F) and thickening of the gallbladder wall (D, E, F).

Ultrasound abdomen demonstrated a distended gallbladder with gallstones and sludge with gallbladder wall thickening. Computerized tomography (CT) of the abdomen showed a distended gallbladder, containing gallstones. There was also thickening of the gallbladder wall, intrahepatic biliary dilatation with mild dilatation of the common bile duct, 11 mm in maximal dimension, and dilated intrahepatic as well as extrahepatic biliary ducts (Figure 1).

**Figure 2:**
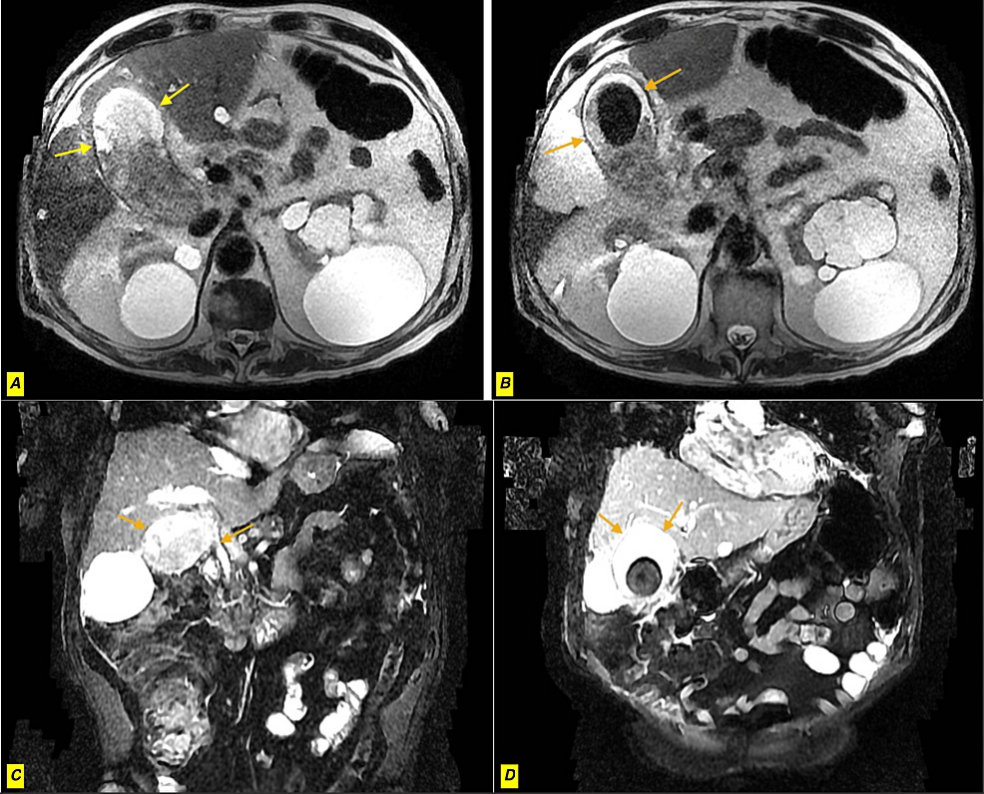
MRCP of the distended gallbladder MRCP: Magnetic resonance cholangiopancreatography Distended gallbladder with multiple gallstones (A), diffuse gallbladder wall thickening, as well as edema with surrounding fat stranding and trace fluid (A, B, C, D). There is a focal narrowing at the superior margin of the common duct bifurcation with the borderline enlarged appearance of intrahepatic biliary ducts (not shown in these selected views).

Magnetic resonance cholangiopancreatography (MRCP) demonstrated a distended gallbladder with multiple gallstones, diffuse gallbladder wall thickening, as well as edema with surrounding fat stranding and trace fluid. At the superior margin of the common duct bifurcation, there was an area of focal narrowing resulting in a borderline enlarged appearance of intrahepatic biliary ducts. No evidence of choledocholithiasis or filling defects along the course of the duct was seen (Figure 2). Nuclear medicine hepatobiliary scan showed normal visualization of the liver, excretion into the biliary tree, common bile duct, and small bowel. Gallbladder was not visualized throughout the examination.

During the hospital stay, the patient was found to manifest symptoms consistent with acute cholecystitis with increased total as well as direct bilirubin and subsequently underwent endoscopic retrograde cholangiopancreatography (ERCP) with biliary sphincterotomy and stent placement. The patient failed medical and interventional endoscopic therapies and was then referred for surgical evaluation. Laparoscopic cholecystectomy was performed. Findings included gangrenous necrotic gallbladder with fundus perforation and adjacent necrotic liver cyst.

On gross examination, the specimen consisted of a resected, previously opened gallbladder, measuring 13 cm in length with a maximal diameter of 5 cm. The serosal surface of the gallbladder was hemorrhagic and edematous, and the hepatic bed was shaggy. The gallbladder contained about 5 cm^3^ of hemorrhagic bile sludges and three green-black gallstones, ranging from 0.2 to 5.5 cm. in greatest dimension. The gallbladder mucosa was hemorrhagic. The thickness of the gallbladder wall was up to 0.6 cm (no gross photos were taken).

**Figure 3:**
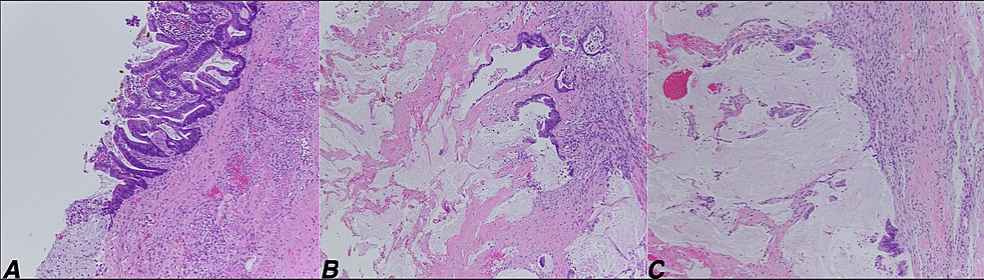
Histopathology images Microscopic photos show hyperchromatic complex tumor glands with intracellular mucin and abundant extracellular mucin (A), and abundant extracellular mucin pools with a few tumor cell clusters (B and C). H&E stain 10X objective magnification.

On microscopic examination, the section showed a moderately differentiated invasive adenocarcinoma. The tumor glands were irregular and hyperchromatic with complex cribriform and papillary growth patterns. Tumor mainly invades the fundus and body of the gallbladder through the muscularis propria into subserosa soft tissue and extracellular mucin pools are identified in tumor tissue. The cystic duct and hepatic bed margins were negative for tumor involvement (Figure 3).

The patient presented with incidental findings of gallbladder malignancy following cholecystectomy. He was informed about the surgical re-exploration for lymphadenectomy, resection of the common bile duct, cystic duct remnant, and gallbladder fossa. However, he deferred treatment at this point and followed up with an oncology outpatient for further management.

## Discussion

Gallbladder cancer (GBC) is the most common biliary tract malignancy with an estimated annual incidence of 1-2 people per 100,000 in the United States [4]. GBC is often aggressive with a poor five-year survival of less than 5%. If stage-adjusted therapy is performed on early-stage GBC, the five-year survival rate can be up to 75% [3]. Although patients with GBC typically present with an advanced stage, a majority of GBC in the United States are diagnosed incidentally post-cholecystectomy for benign diseases such as cholecystitis or cholelithiasis. Because cholecystectomy is one of the most performed general surgery procedures, 0.25-0.7% of cholecystectomy specimens show gallbladder cancer [1]. Patients with incidental gallbladder cancer (IGBC) typically show early-stage disease (T1-T2), therefore, they have a higher five-year survival rate than patients with non-incidental and primary GBC. 

Management of incidental gallbladder cancers can be difficult because the initial cholecystectomy may have resulted in inadequate clearance and nodal evaluation. Because of this, guidelines recommend immediate radical re-resection (IRR) for proper staging and removal of residual disease for tumor stage above T1a [5]. Survival outcomes for IGBC rely on the surgeon’s ability to achieve margin-negative surgery and the presence of residual disease. The success rate of margin-clearing surgery after previous exploration decreases according to the primary tumor T-stage (57% for T2 tumors, 32% for T3, and 16% for T4 respectively) [6]. After GBC has been confirmed from the pathology report, appropriate staging must be conducted prior to IRR. High-quality cross-sectional imaging with computerized tomography (CT) was the most used preoperative imaging modality [7]. In a study of 3,000 patients with GBC conducted over a 15-year span, CT was used in almost 62% of cases, while magnetic resonance imaging (MRI) and positron emission tomography (PET) were used in 6% and 2% of cases respectively [7].

Once staging has been confirmed, re-resection is performed to remove the potential residual regional disease. In an analysis of 684 cases of IGBC that included 124 patients with T1 cancer, an extended re-resection increased the five-year survival up to 68% for T1-IGBC [8]. The analysis demonstrated no advantage for re-resection of T1a (invades lamina propria) cancer, however, IRR for T1b (invades muscular layer) cancer confers a survival benefit from 34% to 75% [8]. The wedge resection of the gallbladder bed with locoregional lymphadenectomy of the hepatoduodenal ligament is the preferred surgical technique for T1b and more advanced cancer [9]. In an analysis of 883 IGBC cases, the five-year overall survival rate of the T2 (invades perimuscular connective tissue) stage is 38% with IRR and 22% without IRR [9]. Furthermore, the analysis concluded a five-year overall survival rate of T3 (invading liver or another organ) to be 18% with IRR and 12% without IRR [5].

## Conclusions

Mucinous adenocarcinoma of the gallbladder is a rare type of gallbladder cancer (GBC) which may be identified as an incidental finding and present as acute cholecystitis or cholelithiasis. It should be considered as a possible differential diagnosis when treating geriatric patients with signs and symptoms of acute cholecystitis and cholelithiasis. Identifying GBC through postoperative histopathological examination is critical to rule out possible malignancy of the gallbladder and determine the type of adenocarcinoma, as well as create the appropriate early surgical treatment and/or medical management protocol according to the best guidelines available. As demonstrated in the present case study, gallbladder adenocarcinoma can closely mimic clinical signs and symptoms of cholecystitis and/or cholelithiasis. The review of the latest research showed that early diagnosis of the GBC and subsequent surgical intervention can play a critical role in prognosis and five-year survival rates.
